# Preliminary Screening of Potential Control Products against *Drosophila suzukii*

**DOI:** 10.3390/insects5020488

**Published:** 2014-06-20

**Authors:** Andrew G. S. Cuthbertson, Debbie A. Collins, Lisa F. Blackburn, Neil Audsley, Howard A. Bell

**Affiliations:** The Food and Environment Research Agency, Sand Hutton, York YO41 1LZ, UK; E-Mails: debbie.collins@fera.gsi.gov.uk (D.A.C.); lisa.blackburn@fera.gsi.gov.uk (L.F.B.); neil.audsley@fera.gsi.gov.uk (N.A.); howard.bell@fera.gsi.gov.uk (H.A.B.)

**Keywords:** *Drosophila suzukii*, control, pesticide

## Abstract

The first recording of *Drosophila suzukii* in the UK occurred in the south of England during August 2012. Since then sticky traps have continued to record the presence of individuals. Several products (both chemical and biological) were investigated for their efficacy against different life-stages of the pest. Both direct and indirect exposure to control products was assessed. Spinosad, chlorantraniliprole and the experimental product, TA2674, showed excellent potential as control agents when used as either a pre- or post-dipping treatment for blueberries with mortalities of 100%, 93% and 98% mortality, respectively, being achieved following pre-treatment. Direct spray application of all products tested had limited impact upon adult flies. Highest mortality (68%) was achieved following direct application of TA2674. Entomopathogenic agents (nematodes and fungi) tested appeared to reduce fly population development (ranges of 34–44% mortality obtained) but would seem unable to eradicate outbreaks. The potential of the tested products to control *D. suzukii* is discussed.

## 1. Introduction

**Figure 1:**
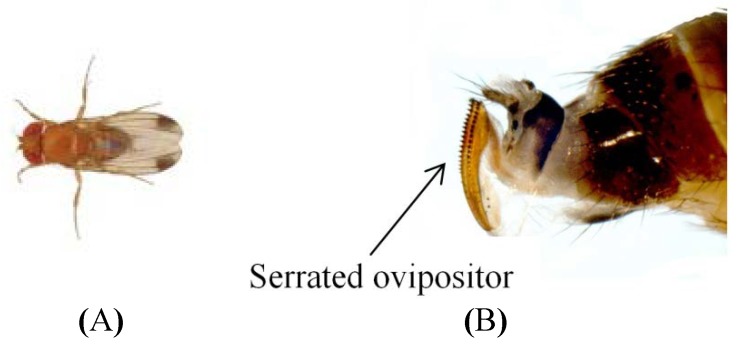
(**A**) Adult male *Drosophila suzukii* (UK Crown Copyright^©^); (**B**) Female serrated ovipositor (Martin Hauser Phycus^©^).

*Drosophila suzukii* Matsumura (Figure 1A) is one of the most serious pests of thin-skinned fruits including blueberry, raspberry, cherry, grape and strawberry [1,2,3,4,5]. Unlike most other Drosophila species *D. suzukii* oviposits and feeds on healthy fruits [6]. *Drosophila suzukii* females possess a serrated ovipositor (Figure 1B) and lay eggs into fresh fruits before harvest [4,7]. Susceptible crops, such as soft-skinned berries are at significant risk from this pest. As a result, a zero tolerance of the fresh fruit market for insect infestation of fruit is resulting in fruit growers having to make major efforts to control *D. suzukii*.

The arrival and spread of *D. suzukii* into major fruit production regions of the northern hemisphere has caused significant economic and sociological impacts [6,8]. This insect originated in eastern Asian countries, including Japan [6]. Since invading Europe and North America as a soft fruit pest in 2008, economic damage has been substantial, with estimated crop losses of up to 50% [4,8,9]. It was estimated that *D. suzukii* damage may lead to $500 million in annual losses in 70 Western USA production areas assuming 30% damage levels [8]. The first recording in Europe was in Spain [6]. Since this time it has steadily moved across Europe, with the first recording of the pest in the United Kingdom (UK) occurring in August 2012 [10]. During 2013, sticky traps continued to record the presence of individuals across England.

Evaluations of insecticides for control of *D. suzukii* have been initiated in most major regions that cover its distribution. These include laboratory bioassays that compared the mortality of flies treated and evaluated in Petri dishes, along with field evaluations where treated plots were sampled for infestation and compared for their control of adult flies. These methods provided important information on the direction of research on control of *D. suzukii* [11]. However, to make effective management decisions about which insecticides are best, it is essential to screen all available products (both commercially available and novel) for their potential to directly control *D. suzukii* and for their ability to prevent larval infestation of fruit by adult flies [12]. Due to damage not being currently significant enough in the UK to allow open field testing of control products; preliminary laboratory screening of several products was undertaken. The aim of the current study was to identify potential products (both UK registered and novel products) for use in the UK against *D. suzukii*.

## 2. Experimental Section

### 2.1. Source of Insects

**Figure 2:**
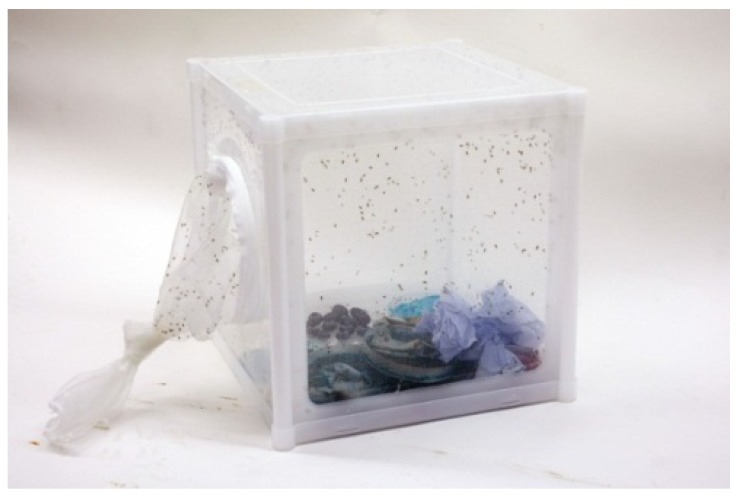
Bug-dorm insect cage containing *Drosophila suzukii* (UK Crown Copyright^©^).

**Figure 3:**
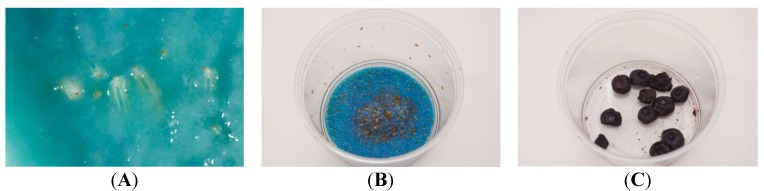
(**A**) *Drosophila suzukii* larvae developing in Drosophila diet (*Blades Biological*, UK); (**B**) Pupae in diet; (**C**) Infested organic blueberries (UK Crown Copyright^©^).

*Drosophila suzukii* used in the experiments originated from wild specimens from Northern Italy, collected in the autumn of 2012. Specimens were imported into the UK under a specific license required for importing non-indigenous invertebrates [13]. A colony was initiated within the secure Insect Quarantine Unit at the Food and Environment Research Agency, York, within bug-dorm (280 mm × 280 mm × 280 mm; *Watkins and Doncaster*, Leominster, UK) insect cages at 25 °C, 65% r.h. and 16:8 h L:D regime (Figure 2). The insects were maintained on a mixture of Drosophila diet (*Blades Biological*, Cowden, UK) and organic blueberries (Figure 3A–C).

### 2.2. Control Products Selected for Investigation

Bioassays were performed using formulated insecticide products. The biological agents tested are also commercially available in the UK. Products (along with their source and active ingredient (a.i.) dose rates tested) were: Coragen (DupPont; chlorantraniliprole; 0.0116g a.i./L), Movento (Bayer CropScience Ltd.; spirotetramat; 0.096 g a.i./L), Calypso (Bayer Cropscience Ltd.; thiacloprid; 0.216 g a.i./L), Conserve (Fargo Ltd.; spinosad; 0.096 a.i./L), Pyrethrum 5EC (Agropharm; pyrethrins; 0.02 g a.i./L), Decis Protech (Bayer CropScience Ltd., deltamethrin; 0.018 g a.i./L), Neem oil (Trifolio-M GmbH; 0.5% solution), Tri-Tek (petroleum oil based product; awaiting UK registration; 2% solution) and a new experimental product (coded: TA2674; 0.017g a.i./L); two entomopathogenic fungi (*Lecanicillium muscarium* as Mycotal (0.1% solution) and *Beauveria bassiana* as Naturalis (0.3% solution)); three entomopathogenic nematodes (*Steinernema carpocapsae*, *S. feltiae*, *S. kraussei*; all tested at 10,000 Infective Juveniles per mL).

### 2.3. Laboratory Bioassays to Investigate Effectiveness of Chemical Control Products

Blueberries (400 in total) were infested for 72 hours within four bug-dorm cages (100 berries per cage) each containing approximately 70 mixed-sex adult *D. suzukii*. Following this infestation period the blueberries were cleaned of adult flies and equal numbers were randomly dipped (full emersion) in field-rate concentrations of the following products: chlorantraniliprole, spirotetramat, thiacloprid, spinosad, pyrethrins, deltamethrin, Neem oil, Tri-Tek and TA2674. A water treatment acted as control. 40 berries were dipped in each control product. After dipping, berries were placed into 10 cm diameter ventilated plastic deli-pots and placed into a Controlled Environment (CE) cabinet and incubated for 10 days at 25 °C. The pots were then assessed for presence of adult flies and the berries dissected to inspect for presence of larvae and/or pupae development. 

### 2.4. Investigating Impact of Entomopathogenic Nematodes and Fungi on *Drosophila suzukii* Emergence

Berries were again infested for 72 hours (264 in total) in bug-dorm cages each containing approximately 70 adult mixed-sex flies. Forty four blueberries were then selected at random per treatment and, after full emersion in standard formulations of the treatment products (*L. muscarium*, *B. bassiana*, *S. carpocapsae*, *S. feltiae*, *S. kraussei*) were incubated in a CE cabinet for 10 days at 25 °C. All larvae, pupae and adult flies that developed were counted. Berries were dissected for signs of larvae in treated dishes. Equal numbers of berries dipped in water acted as controls.

### 2.5. Impact of Direct Application of Chemical Products on Adult *Drosophila suzukii*

Spinosad, chlorantraniliprole and TA2674 were applied directly at standard field rates against equal numbers of male and female adult *D. suzukii* using an automatic-load Potter precision laboratory spray tower. Following treatment adult flies were maintained at 25 °C and supplied with standard Drosophila medium as a food source within ventilated plastic deli-pots. Mortality was assessed following 24 and 48 hours. Direct application of all biological products (nematodes and fungi) was also undertaken and mortality was assessed after 7 days. For all treatments there were 5 replicates of 10 adult flies (5 male and 5 female; 50 adults in total). Individuals sprayed with water acted as controls.

### 2.6. Potential of Products to Act as Oviposition Deterrents

To investigate the potential of the most efficient products from the current study to act as oviposition deterrents, blueberries were first dipped in the standard dose rates of Spinosad, Coragen and TA2674 (100 berries per product). They were then placed on 9 cm diameter Petri dishes (10 berries per dish) and allowed to air dry for 2 hours. Berries dipped in water acted as controls. The petri dishes were then placed into 10cm diameter plastic deli-pots with ventilated lids. Ten adult *D. suzukii* (5 males and 5 females) were then introduced to the berries contained within the deli-pots. All were maintained in a CE cabinet at 25 °C. Mortality of the introduced adult flies was assessed over 48 hours. The berries were maintained at 25 °C for a further 10 days at which time adult fly emergence was determined. Following this, the berries were dissected and examined for presence of any remaining larvae and/or pupae development.

### 2.7. Data Analysis

Data was statistically analysed where appropriate. Treatments were compared against the control. For analysis the numbers for each life stage in each individual treatment were combined. Assuming normality and constant variance, Analysis of Variance (ANOVA) was used to test any significant difference.

## 3. Results and Discussion

**Figure 4:**
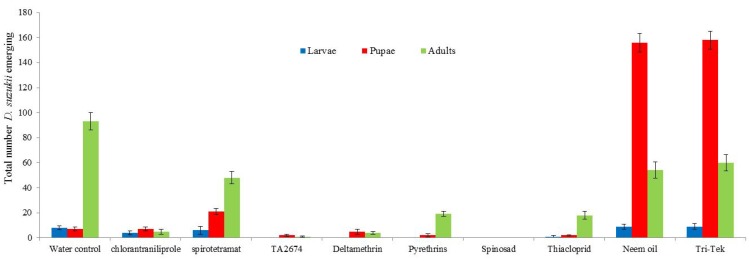
Impact of treatments on emergence of *Drosophila suzukii* from infested blueberries (assessed after 10 days incubation at 25 °C). Total numbers from 40 berries per treatment.

Spinosad, deltamethrin and the new experimental product (TA2674) proved most effective in controlling *D. suzukii* (Figure 4). Spinosad caused complete mortality of *D. suzukii*; no flies emerged from treated berries. Deltamethrin, TA2674, pyrethrins and thiacloprid all had significantly less *D. suzukii* numbers emerging from treated berries compared to the water control (*p* < 0.01). The products Neem oil and Tri-Tek would appear to have delayed population development of the flies as larger numbers of pupae were recorded in these treatments after 10 days incubation.

**Figure 5:**
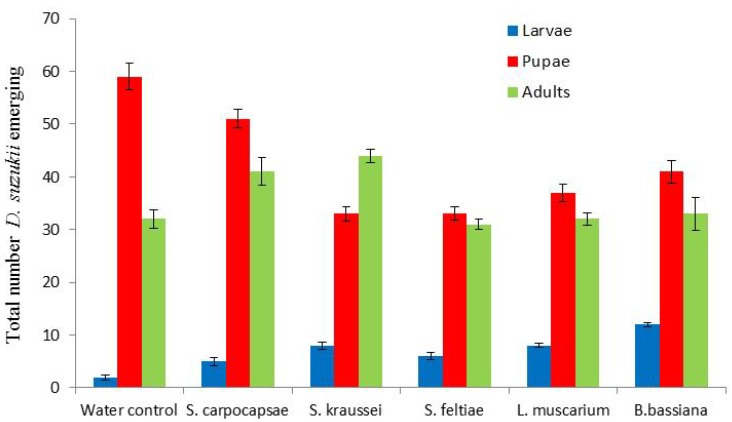
Impact of dipping infested berries in entomopathogenic nematodes and fungi on resulting fruit fly emergence following 10 days incubation at 25 °C. Total numbers from 44 berries per treatment.

Both the nematodes and fungi would appear to cause population decreases of *D. suzukii* (Figure 5). However, neither would seem to have the potential to eradicate *D. suzukii*. There was no marked impact on fly emergence, with populations developing as normal (Figure 5). There was no significant difference between the individual control agents or the water control (*p* > 0.05).

**Figure 6:**
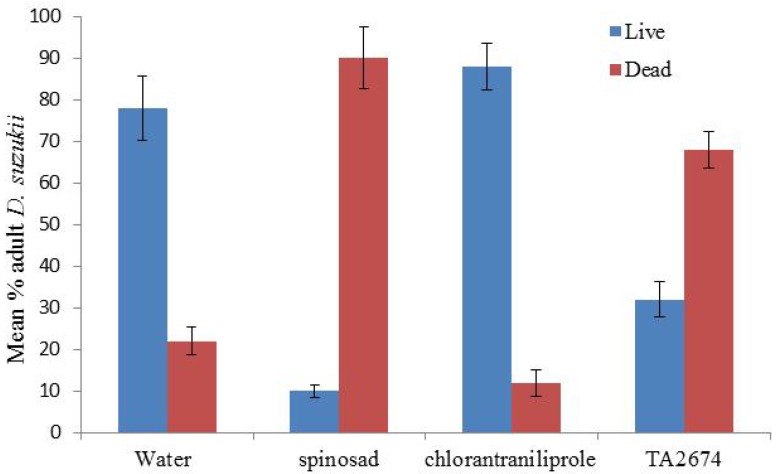
Impact of direct application of chemical control products against adult *Drosophila suzukii*. Mortality assessed after 48 hours (50 flies per treatment).

**Figure 7:**
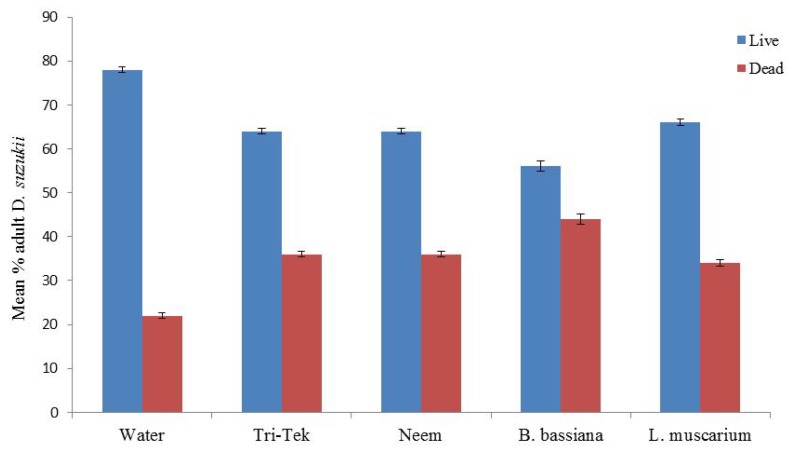
Impact of direct application of alternative products and entomopathogenic fungi against adult *Drosophila suzukii*. Mortality assessed after 7 days (50 flies per treatment).

Direct application of all products had limited mortality on adult *D. suzukii*. Spinosad proved the best with 90% mortality being achieved, significantly higher than the water control (*p* < 0.001) (Figure 6). Direct application of all the ‘green’ products (Neem oil, Tri-Tek and the entomopathogenic agents) all caused significantly higher mortality than the water control (*p* < 0.05) (Figure 7). *Beauveria bassiana* caused 44% adult mortality after 7 days (*p* < 0.01). However, the entomopathogenic control products would not seem to be effective in controlling *D. suzukii* numbers as the next generation of larvae were already coming through in the feeding media following one week (Figure 3A).

**Figure 8:**
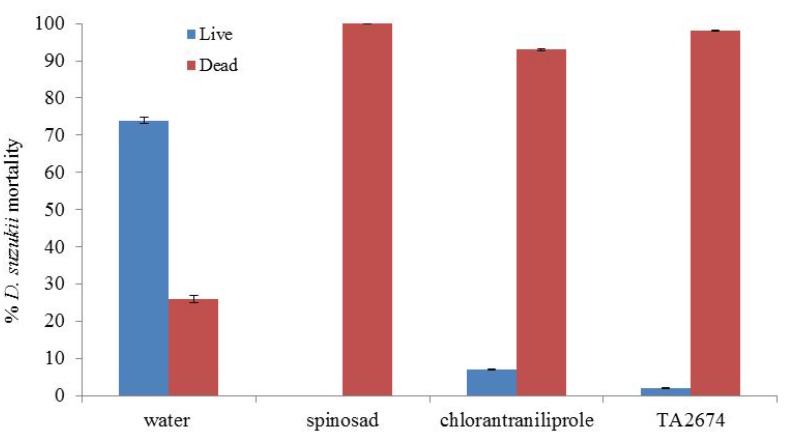
Mortality observed of adult *Drosophila suzukii* flies following exposure to pre‑treated blueberries. Mortality assessed after 48 hours (100 flies per treatment).

**Figure 9:**
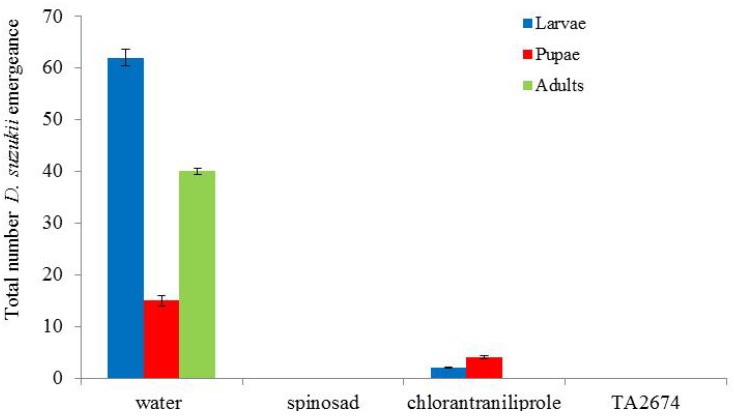
Impact of pre-treating blueberries with chemical insecticides on subsequent *Drosophila suzukii* infestation and development (assessed after 10 days incubation at 25 °C).

In assessing the potential of the most efficient products to act as oviposition deterrents, following 48 hours after the addition of the adult flies, 100 and 98% mortality was recorded in the spinosad and TA2674 treatments, respectively (Figure 8). Subsequently, following a further incubation of 10 days, no flies developed from these berries (Figure 9). Both spinosad and TA2674, therefore, provided complete protection from *D. suzukii* following the pre-treatment of fresh berries. Larvae, pupae and adults developed in the control berries as expected.

This preliminary study has shown that currently available insecticides for use against *D. suzukii* in the UK can provide control, and in the case of spinosad and the experimental product (TA2674) eradication. Spinosad, under laboratory conditions, has been shown to offer excellent control of *D. suzukii*. The results demonstrate that when uninfected berries are dipped in spinosad, and indeed TA2674, adult flies are prevented from ovipositing or if eggs are deposited the subsequent larvae/pupae do not develop; death occurs. In the current study we only tested the selected products’ efficacy on blueberry fruit and, as *D. suzukii* readily feeds on various soft fruits [14], product efficacy still needs further testing on different host fruits. Our laboratory experiments provided little data for the support of the use of entomopathogenic fungi or nematodes as control agents against *D. suzukii* on blueberries. This, however, is in contrast to certain high levels of control obtained by similar products based on *B. bassiana*, reported by other studies [15,16]. This highlights the need to screen all available species and strains of fungi for their efficacy against a given pest species. Tri-Tek, a petroleum oil based product, proved disappointing in the current study. This product has shown excellent potential for incorporation into pest management strategies against adult whitefly, *Bemisia tabaci* [17]. This study [17] reported that Tri-Tek acted through asphyxiation by trapping the flies while wet.

Laboratory data generally becomes more variable when treatments are transferred to the field. The findings from the current study, obtained under controlled conditions, must be tested on a broader scale before firm conclusions can be drawn. It has been shown that the efficacy of most insecticidal treatments is reduced greatly after exposure to just over 2 cm of rain and that after one week following treatment adult (*D. suzukii*) mortality is often not significantly different from the untreated controls for most insecticides that are exposed to rain [11]. In an outdoor situation, should berry protection be required but rain is forecast, the effectiveness of insecticides in this situation could possibly be enhanced by the addition of adjuvants to reduce loss due to rain.

## 4. Conclusions

*Drosophila suzukii* presents a real challenge to the UK horticultural industry. Containment and/or eradication of this pest will prove difficult. Data from our trials indicates that spinosad along with chlorantraniliprole and the experimental product TA2674 show excellent potential as control agents of *D. suzukii* when used as either a pre or post-dipping treatment. None of the products tested provided complete mortality following direct application to adults, and larvae were seen to be much more susceptible following berry dipping. The biological agents (fungi and nematodes) caused reductions in population numbers of *D. suzukii* but are unlikely to control/eradicate populations. However, they should prove easy to incorporate into existing invertebrate control programmes as shown in other pest control strategies [18]. Maintaining coverage of fruit clusters will be essential for effective protection against *D. suzukii*.

## References

[1] Sasaki M., Sato R. (1995). Bionomics of the cherry Drosophila, *Drosophila suzukii* Matsumura (Diptera: Drosophilidae) in Fukushima Prefecture, 3: Life cycle. Annu. Rep. Plant Prot. N. Jpn..

[2] Lee J.C., Bruck D.J., Curry H., Edwards D., Haviland D.R., van Steenwyk R.A., Yorgey B.M. (2011). The susceptibility of small fruits and cherries to the spotted-wing drosophila, *Drosophila suzukii*. Pest Manag. Sci..

[3] Lee J.C., Bruck D.J., Dreves A.J., Ioriatti C., Vogt H., Baufeld P. (2011). In Focus: Spotted wing drosophila, *Drosophila suzukii*, across perspectives. Pest Manag. Sci..

[4] Walsh D.B., Bolda M.P., Goodhue R.E., Dreves A.J., Lee J., Bruck D.J., Walton V.M., O’Neal S.D., Zalom F.G. (2011). *Drosophila suzukii* (Diptera: Drosophilidae): Invasive pest of ripening soft fruit expanding its geographic range and damage potential. Int. J. Pest Manag..

[5] Bellamy D.E., Sisterson M.S., Walse S.S. (2013). Quantifying host potentials: Indexing postharvest fresh fruits for spotted wing drosophila, *Drosophila suzukii*. PLoS One.

[6] Calabria G., Máca J., Bächli G., Serra L., Pascual M. (2012). First records of the potential pest species *Drosophila suzukii* (Diptera:Drosophilidae) in Europe. J. Appl. Entomol..

[7] Sasaki M., Abe N. (1993). Occurrence of Drosophila in the cherry orchards (1) Species and the seasonal prevalence obtained by the bite trap. Annu. Rep. Plant Prot. N. Jpn..

[8] Goodhue R.E., Bolda M., Farnsworth D., Williams J.C., Zalom F.G. (2011). Spotted wing drosophila infestation of California strawberries and raspberries: Economic analysis of potential revenue losses and control costs. Pest Manag. Sci..

[9] Cini A., Ioriatti C., Anfora G. (2012). A review of the invasion of *Drosophila suzukii* in Europe and a draft research agenda for integrated pest management. Bull. Insectol..

[10] Cuthbertson A.G.S., Collins D.A., Blackburn L.F., Bell H.A. Screening products for control of *Drosophila suzukii*. Presented at the 8th Meeting of the IOBC-WPRS Working Group “*Integrated Plant Protection in Fruit Crops*”, Sub Group “Soft Fruits”: “*Workshop on Integrated Soft Fruit Production*”.

[11] Bruck D.J., Bolda M., Tanigoshi L., Klick J., Kleiber J., DeFrancesco J., Gerdeman B., Spitler H. (2011). Laboratory and field comparisons of insecticides to reduce infestation of *Drosophila suzukii* in berry crops. Pest Manag. Sci..

[12] Van Timmeren S., Isaacs R. (2013). Control of spotted wing drosophila, *Drosophila suzukii*, by specific insecticides and by conventional and organic crop protection programs. Crop Prot..

[13] Marris G., Cuthbertson A.G.S., Mathers J.J., Blackburn L.F. (2010). Containing the small hive beetle for research purposes. Bee Craft.

[14] Steffan S.A., Lee J.C., Singleton M.E., Vilaire A., Walsh D.B., Lavine L.S., Patten K. (2013). Susceptibility of cranberries to *Drosophila suzukii* (Diptera:Drosophilidae). J. Econ. Entomol..

[15] Brust J. Spotted Wing Drosophila Management in Maryland Small Fruit. http://www.caf.wvu.edu/kearneysville/SWD/Spotted_Wing_Drosophila-Jerry_Brust.pdf.

[16] Gargani E., Tarchi F., Frosinini R., Mazza G., Simoni S. (2013). Notes on *Drosophila suzukii* Matsumura (Diptera Drosophilidae): Field survey in Tuscany and laboratory evaluation of organic products. Redia-Giornale Di Zoologia.

[17] Cuthbertson A.G.S., Buxton J.H., Blackburn L.F., Mathers J.J., Robinson K., Powell M.E., Fleming D.A., Bell H.A. (2012). Eradicating *Bemisia tabaci* Q on poinsettia plants in the UK. Crop Prot..

[18] Cuthbertson A.G.S. (2013). Update on the status of *Bemisia tabaci* in the UK and the use of entomopathogenic fungi within eradication programmes. Insects.

